# Distributing transmitters to maximize population-level representativeness in automated radio telemetry studies of animal movement

**DOI:** 10.1186/s40462-022-00363-0

**Published:** 2023-01-04

**Authors:** Juliet S. Lamb, Pamela H. Loring, Peter W. C. Paton

**Affiliations:** 1 New York Division, The Nature Conservancy, Cold Spring Harbor, New York, NY USA; 2 Division of Migratory Birds, U.S. Fish and Wildlife Service, North Atlantic-Appalachian Region, Charlestown, RI USA; 3 University of Rhode Island, Kingston, RI USA

**Keywords:** *Charadrius melodus*, Common tern, Motus, Piping plover, Power analysis, Offshore wind, Sample size, Shorebird, Seabird, *Sterna hirundo*

## Abstract

Telemetry is a powerful and indispensable tool for evaluating wildlife movement and distribution patterns, particularly in systems where opportunities for direct observation are limited. However, the effort and expense required to track individuals often results in small sample sizes, which can lead to biased results if the sample of tracked individuals does not fully capture spatial, temporal, and individual variability within the target population. To better understand the influence of sampling design on results of automated radio telemetry studies, we conducted a retrospective power analysis of very high frequency (VHF) radio telemetry data from the Motus Wildlife Tracking System for two species of birds along the United States Atlantic coast: a shorebird, the piping plover (*Charadrius melodus*), and a nearshore seabird, the common tern (*Sterna hirundo*). We found that ~ 100–150 tracked individuals were required to identify 90% of locations known to be used by the tracked population, with 40–50 additional individuals required to include 95% of used locations. For any number of individuals, the percentage of stations included in the sample was higher for common terns than for piping plovers when tags were deployed within a single site and year. Percentages of stations included increased for piping plovers when birds were tagged over multiple sites and, to a lesser extent, years, and increased with average length of the tracking period. The probability that any given receiver station used by the population would be included in a subsample increased with the number of birds tracked, station proximity to a migratory stopover or staging site, number of receiving antennas per station, and percentage of the tracked population present. Our results provide general guidance for the number and distribution of tagged birds required to obtain representative VHF telemetry data, while also highlighting the importance of accounting for station network configuration and species-specific differences in behavior when designing automated radio telemetry studies to address specific research questions. Our results have broad applications to remotely track movements of small-bodied migratory wildlife in inaccessible habitats, including predicting and monitoring effects of offshore wind energy development.

**Supplementary Information:**

The online version contains supplementary material available at 10.1186/s40462-022-00363-0.

## Background

Understanding wildlife habitat use and movement ecology often requires using transmitters to detect individuals in areas where opportunities for direct observation are limited. In cases where habitat characteristics or behavior of focal species present challenges to observing wildlife, individual tracking can be used to fill gaps in understanding of current distribution and predict responses to future conditions. Due to the high costs of tracking devices and welfare concerns related to the effects of capture and tagging, however, studies of animal movement often involve inferring population-level movement patterns from a small sample of individuals. Thus, it is crucial that the individuals selected for tracking effectively represent underlying individual, spatial, and interannual variation in movement patterns in the target population to avoid biasing the results [1].

Despite the importance of sampling design in telemetry studies, power analyses to inform study design remain relatively rare [2]. Sampling parameters are dictated by the cost, size, and technological limitations of tracking devices, rather than designed to maximize statistical power of the results to detect target patterns of movement [1, 2]. Nevertheless, accumulation of tracking data over time has created opportunities to synthesize existing data and assess how the number and distribution of sampled individuals affects detection of variation and population-level inferences [e.g., 3,4]. Such retrospective studies have generally focused on active transmitters that actively collect, store, and/or upload location data via satellite networks. Sampling considerations for passive transmitters, such as radio frequency identification (RFID), VHF, ultra high frequency (UHF), and acoustic units, whose detection depends on receiving antennas, have received comparatively less attention.

Most passive transmitters have several key advantages over GPS or satellite transmitters, including small sizes and costs. However, they also have unique sampling considerations that deserve attention. While detection of global positioning system (GPS) and ARGOS satellite transmitters depend on programming decisions such as sampling frequency, which affects the temporal resolution of data [5], their spatial coverage is global. In contrast, spatial coverage of locating passive transmitters is limited by the range of external receiving antennas to detect individuals passing through a focal area. Therefore, assessment of the effects of sampling design on distributional data derived from networks of receiving stations is required to identify factors affecting representativeness of tracked populations and inform future studies.

Given the challenges of directly observing wildlife movements in coastal and offshore marine environments, individual tracking is a vital component of understanding wildlife distributions in marine systems and predicting effects of future change. In North America, offshore wind energy development is nascent but rapidly advancing. Installations generating at least 16 gigawatts (GW) of energy are currently slated for development off the U.S. Atlantic coast, contributing to a national target of 30 GW by 2030 [6]. This ambitious development schedule has resulted in a need to rapidly acquire baseline data on wildlife presence and movements within and between offshore wind lease areas, as well as to develop robust monitoring protocols for measuring and determining the effects of offshore wind energy development on wildlife species of conservation concern [7]. Offshore wind stakeholders are recommending installation of fixed antenna arrays in offshore wind planning areas to detect passive transmitters on wildlife (i.e., both VHF and UHF transmitters), particularly small-bodied birds and bats, before, during, and after construction. However, the utility of antennas to detect movement patterns is highly dependent on the configuration of the antenna array, including the number and distribution of antenna stations and the heights of antennas relative to flight heights of target species [8]. Moreover, if an insufficient number of individuals are tagged with transmitters, or if the population selected for transmitter deployment does not represent the population using the location or route of interest, then use of specific sites (such as an offshore wind energy area) could be missed. To date, however, lack of information on the number and distribution of transmitters required for detection of baseline occupancy patterns has precluded the development of guidance for appropriate transmitter deployment.

To address this information gap, we tested the effect of sample size on the detection probability of free-flying VHF-tracked birds by a fixed array of coastal receiving stations along the mid-Atlantic coast of the United States in coordination with the Motus Wildlife Tracking System, an international radio telemetry network that automatically detects and identifies organisms with VHF transmitters and stores detection data for subsequent download and analysis [9]. Few receiving stations are currently deployed in the offshore environment; however, existing data from onshore stations can be used as a starting point to assess effects of number and distribution of transmitters on detection patterns and begin developing sampling guidelines. We focused our analysis on two representative coastal species with different migratory patterns and habitat requirements: a shorebird (piping plover *Charadris melodius*) and a nearshore seabird (common tern *Sterna hirundo*). Our objectives were to (1) subsample existing data to determine the number of tagged individuals required to represent population-level occupancy patterns for each species across Motus stations; (2) assess whether distributing transmitters among different combinations of tagging sites and years improved probability of detecting occupied sites; and (3) evaluate how the distance of receiving stations from the tagging site affects detection probability across sample sizes, tag distributions, and species.

## Methods

### Data collection

#### Study species

Piping plovers are small-bodied (43–63 g) migratory shorebirds. On the Atlantic coast of North America, where the species is federally listed as Threatened, their breeding range extends from the Canadian Maritimes to North Carolina, and their wintering range occurs from the mid-Atlantic United States to the Gulf of Mexico and the Caribbean [10]. In coastal areas, piping plovers typically nest on open sandy beaches and forage on a variety of invertebrates along the shoreline and intertidal zone [10].

Common Terns are globally-distributed, mid-sized (110–145 g) seabirds that breed throughout the Northern Hemisphere and winter along tropical and subtropical coasts [11]. Along the northern Atlantic coast of North America, Common Terns nest colonially on islands and barrier beaches from Newfoundland and Labrador, Canada to South Carolina, USA [11]. During their northerly migration, common terns have been observed flying over open ocean more than 50 km off the coasts of Virginia and Massachusetts [12–14].

#### Capture and tagging

We tracked movements of piping plovers and common terns using uniquely coded VHF transmitters (“nanotags”, Lotek Wireless, Ontario, Canada) at focal breeding sites in southern New England. We were interested in tracking breeding-season and fall migratory departure movements of these species along the U.S. Atlantic Coast and adjacent OCS waters from Cape Cod, Massachusetts in the north to Back Bay, Virginia to the south. As of May 2022, this area contains 25 BOEM Renewable Energy Commercial Lease Areas and one Research Lease Area [6].

**Fig. 1:**
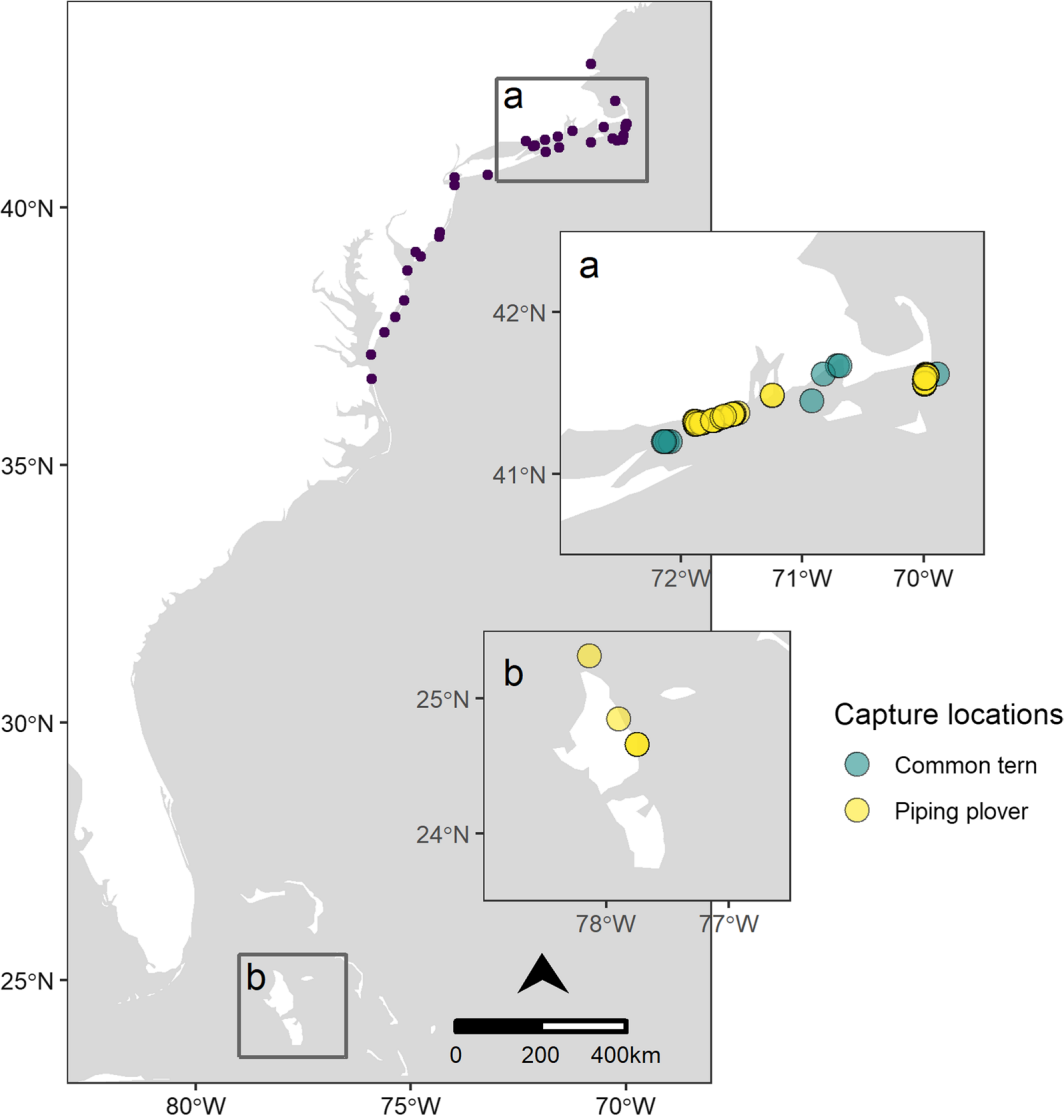
Motus antenna locations along the Atlantic coast of North America (black dots) that detected either piping plovers or common terns in this study and were continuously operational between 2014 and 2017. Inset maps show relative locations of **a** New England tagging sites for piping plovers (yellow; 2015–2017) and common terns (teal; 2014–2017); and **b** Bahamas tagging locations for piping plovers (yellow; 2017 only)

We tagged piping plovers between 2015 and 2017 in two principal breeding locations: southeastern Massachusetts (Monomoy Island and South Beach), and the southern coast of Rhode Island (Fig. 1). Monomoy and South Beach account for ~ 9% of the Massachusetts population of piping plovers, while focal sites in Rhode Island supported at least one third of nesting pairs monitored in the state [14]. From 9 May through 27 June, we captured adult plovers at their nest sites during the egg incubation period using funnel traps. A small number (*n* = 10) of piping plovers were tagged in the Bahamas during March 2017 with the aim of tracking northbound migratory movements. Tagging sites were in the northwestern Bahamas on North Andros (Young Sound and Kamalame Cay) and the Joulter Cays. Plovers were captured in diurnal foraging areas using drop nets. All piping plovers were fitted with Lotek NTQB-4-2 (1.1 g; 12 × 8 × 8 mm; < 3% body mass) nanotags with 16.5-cm antennas. Tags were attached by clipping a small area of feathers from the interscapular region and gluing the tag to the feather stubble and skin with a cyanoacrylate gel adhesive [8].

Between 2014 and 2017, we tagged common terns at several breeding colonies across the same southern New England range (Fig. 1): at Great Gull Island, NY, in eastern Long Island Sound; at Monomoy Island (2014); and at three colonies in Buzzard’s Bay, Massachusetts (Bird, Ram, and Penikese Islands; 2016–2017). From 9 June to 12 July, staff at nesting colonies used walk-in treadle traps to capture adult common terns at their nests, within approximately 1–5 days of their eggs hatching. Common terns were fitted with waterproofed Lotek NTQB-4-2 nanotags with 1-mm tubes at the front and back of the transmitter, bringing the total tag weight to 1.5 g. The transmitter and attachment materials weighed < 2% of the body mass of tagged terns. We attached nanotags to the dorsal inter-scapular region using cyanoacrylate adhesive and two sutures (Prolene: 45-cm length, 4.0, BB taper point needle, catalog # 8581H) inserted subcutaneously and secured to the end-tubes of the transmitter [14].

All transmitters were programmed to continuously transmit on a shared frequency of 166.380 MHz from activation through the end of battery life. Times between transmissions (burst intervals) were specific to each transmitter and ranged from 4 to 6 s. All transmitters were uniquely identifiable on the shared frequency using a combination of an encoded tag ID and known burst intervals in coordination with the Motus Wildlife Tracking System [9]. The expected life of the NTQB-4-2 nanotags ranged from 146 days (4 s burst interval) to 187 days (6 s burst interval). For each tracked individual, we tallied the total number of transmissions received and calculated the duration of transmission by subtracting the deployment date from the date of last transmission received.

#### Automated telemetry stations

We obtained signals of tagged birds using an array of land-based automated radio telemetry stations (i.e., stations that automatically scan for organisms with uniquely-coded VHF transmitters and record detections; hereafter, stations) throughout the study area (Additional file 1: Table S1). Typical station specifications consisted of a 12.2-m tall radio antenna mast supporting 4–6, 9-element (3.3 m) Yagi antennas mounted in a radial configuration at 60-degree intervals. Antennas were connected to ports on a receiving unit (Lotek SRX-600 or 800, Lotek Wireless, Ontario, Canada) via coaxial cable (TWS-200). Each receiving station was operated 24 h per day using one 140-W solar panel and two 12-V deep- cycle batteries. When tagged birds were within detection range, the receivers automatically recorded transmitter ID number, date, time stamp, antenna (defined by monitoring station and bearing), and signal strength value of each detection. All raw data and metadata were uploaded to the Motus Wildlife Tracking System for processing [9]. We used the ‘motus’ package in R to download and filter detection data using default criteria [15].

### Data analysis

#### Bootstrapped inclusion values

We began by using detection radius estimates and active dates provided on the Motus website [16] to combine any receiving stations that were placed in identical locations and eliminate any stations or station groups that were not active during the full study period. We then used a bootstrapping approach to assess how well different subsamples of the population represented occurrence patterns of un-sampled individuals [17, 18]. This involved repeatedly selecting random subsamples of *n* individuals from the overall sample *N *and calculating the inclusion value. We defined the inclusion value as the proportion of stations used by the subsampled population that were also used by the remaining individuals not included in the subsample. We conducted all analyses in R [19].

For each sample size *n* up to a maximum of *N *− 1, we repeated the process 50 times and calculated the mean (µ) and standard deviation of the inclusion value for a given sample size (*I*_*n*_) over the 50 random subsamples. We then fitted a non-linear model to the means of the bootstrapped samples of the form µ*I*_*n*_ = a*n*/1 + b*n*, where a and b represent numeric coefficients. We estimated parameter values via non-linear least squares estimation, with starting values of a = 1 and b = 0.1. We used the modeled relationship between the inclusion value and sample size to identify sample sizes required to achieve inclusion at three different levels—80%, 90%, and 95%—that approximately correspond to confidence intervals commonly used to detect significant effects. After fitting a non-linear function to the data, we calculated the representativeness for any given sample size (*R*_*n*_) by dividing the projected inclusion value for that subsample (*I*_*n*_) by the asymptote of the fitted non-linear function (*I*_*max*_): *R*_*n*_ = *I*_*n*_/*I*_*max*_. We also calculated the representativeness of each capture site as the asymptote for that capture site alone, divided by the asymptote across the full population (*R(capture site)* = *I*_*max*_*(capture site)*/*I*_*max*_*(population)*).

We calculated representativeness across different combinations of tagging sites and years by constraining our random draws to specific number of tagging sites (1–2) or years (1–3), then comparing the inclusion values from each combination of sites and years with the asymptotic inclusion value *I*_*max*_ for the overall sample. Since the Bahamas were only sampled in one year for piping plovers, we removed individuals captured at this site from analysis of site-year effects. Similarly, since common terns were only captured at one site (GGI) in 2015, we removed this year from analysis of site-year effects but retained all years with two distinct capture sites.

Finally, for piping plovers, we also assessed the detection probabilities of detecting at individual receiving stations by calculating the probability that each used station would be included in the subset of stations used by a bootstrapped subsample of *n* individuals (*T*_*n*_). Since common tern sampling was designed to detect individuals during post-breeding staging but not during migration [20], we expected station characteristics to play a limited role in station-specific detection rates for this species; we therefore did not assess this metric for common terns.

#### Modeling

We fit generalized additive models (GAMs) with residual maximum likelihood [21] using the mgcv R package [22] to determine the effects of dividing any given sample size across multiple tagging sites or years on both inclusion values and their variability. We modeled either the mean or the standard deviation of the raw bootstrapped inclusion values (response) as a function of number of transmitters (smoothed predictor), number of tagging sites (categorical predictor), number of years (categorical predictor), average duration of transmission (i.e., number of days between tag deployment and last detection; smoothed predictor), and average number of detections per individual (smoothed predictor). We identified a predictor as significant if its 95% confidence interval (CI) did not overlap zero. To assess factors affecting inclusion probabilities for used stations, we modeled means and standard deviations of station-specific inclusion probabilities *T*_*n*_ as a function of number of transmitters, distance to tagging site, number of receiving antennas, antenna altitude, and number of detections at the station (smoothed predictors).

## Results

The piping plover sample included 129 individuals with at least one detection: 6 plovers tracked from the Bahamas (2017 only), 61 plovers tracked from Rhode Island (2015: *n* = 21; 2016: *n* = 17; 2017: *n* = 21), and 62 plovers tracked from Massachusetts (2015: *n* = 21; 2016: *n* = 18; 2017: *n* = 23) (Fig. 1). An additional 31 piping plovers (Rhode Island: *n* = 14; Massachusetts: *n* = 13; Bahamas: *n* = 4), or 20% of the total sample, were tagged but never detected.

**Fig. 2:**
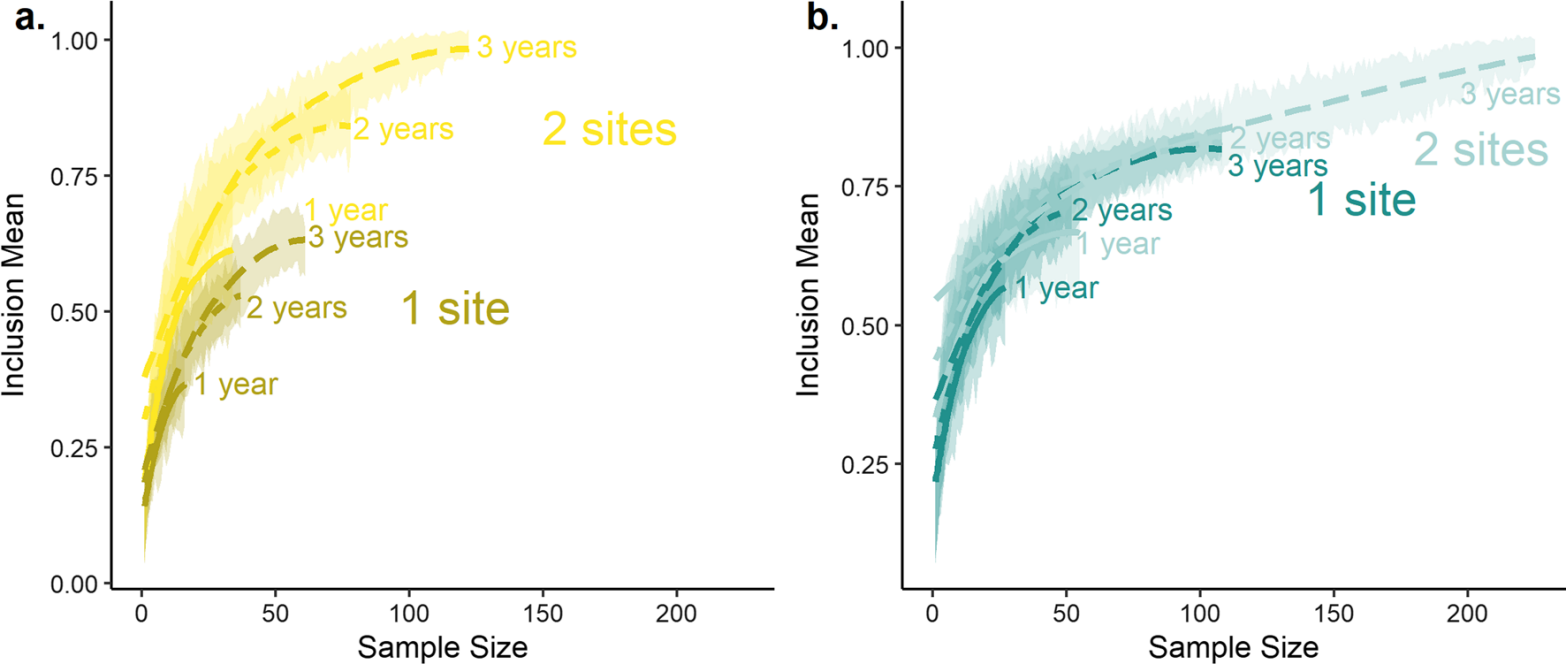
Bootstrapped inclusion values by sample size for tagged **a** piping plovers (2015–2017), and **b** common terns (2014–2017). Lines indicate smoothed mean values obtained from 50 bootstrapped samples for each sample size, and shaded areas indicate standard deviations of mean inclusion values

For all tagging sites combined, a sample size of 90 plovers was required to achieve 90% representativeness of the unsampled population, with a maximum possible representativeness of 96% (Table 1). For each unique tagging site or combination of sites within the sample, a mean sample of 38 individuals (range = 34–51) was generally sufficient to represent 80% of stations used by unsampled individuals in that subgroup, and 81 individuals (range = 64–128) were required to achieve 90% representativeness (Table 1). The representativeness of any single tagging site ranged from 21–65%, and from 68–93% for two sites (Table 1). For any given sample size, representativeness was higher for two tagging sites than for one and increased with the number of years sampled (Fig. 2a).

**Table 1:** Species-specific site- and population-level representativeness (R) for individuals tracked from each site or combination of sites, and projected sample sizes needed to achieve 80–95% representativeness at the population and site levels

	Site	*n*	Tagging site				Population			
			*R*	80%	90%	95%	*R*	80%	90%	95%
*Piping plover*										
One tagging site	Rhode Island (RI)	61	87%	37	81		59%			
	Massachusetts (MA)	62	87%	36	75		65%			
	Bahamas	6	27%	51	128		21%			
Two tagging sites	RI + Bahamas	67	87%	34	73		68%			
	MA + Bahamas	68	85%	38	83		81%	129		
	RI + MA	123	95%	35	66	94	93%	43	94	121
Three tagging sites	All	129	96%	34	64	90	96%	42	90	125
*Common tern*										
One tagging site	Great Gull Island (GG)	140	98%	24	46	84	81%			
	Buzzards Bay (BB)	57	93%	29	50		76%			
	Monomoy Island (MY)	65	88%	41			47%			
Two tagging sites	GG + MY	205	98%	29	58	105	81%	136		
	BB + MY	122	94%	55	95		83%	84		
	GG + BB	197	97%	36	88	123	94%	58	124	164
Three tagging sites	All	262	97%	43	109	155	97%	76	145	208

The common tern sample included 262 individuals with at least one detection: 140 terns tracked from Great Gull Island (2014: *n* = 51; 2015: *n* = 31; 2016: *n* = 30; 2017: *n* = 28), 57 terns tracked from Buzzards Bay (2016: *n* = 29; 2017, *n* = 28), and 65 terns tracked from Monomoy in 2014 (Fig. 1). Almost all (98%) of tagged individual terns were detected, with only four individuals from Great Gull (*n* = 2) and Buzzards Bay (*n* = 2) not detected. For all tagging sites combined, a sample size of 145 terns was required to achieve 90% representativeness of the unsampled population, with a maximum representativeness of 97% (Table 1). For each unique site or combination of sites, a mean sample of 33 individuals (range = 24–43) was generally sufficient to represent 80% of station locations used by unsampled individuals in that subgroup, and 73 individuals (range = 46–109) were required to achieve 90% representativeness (Table 1). The maximum population-level representativeness for a single tagging site ranged from 54–86%, and from 82–94% for two sites (Table 1). For any given sample size, representativeness was similar regardless of the number of sites sampled but increased with additional sampling years (Fig. 2b).

**Fig. 3:**
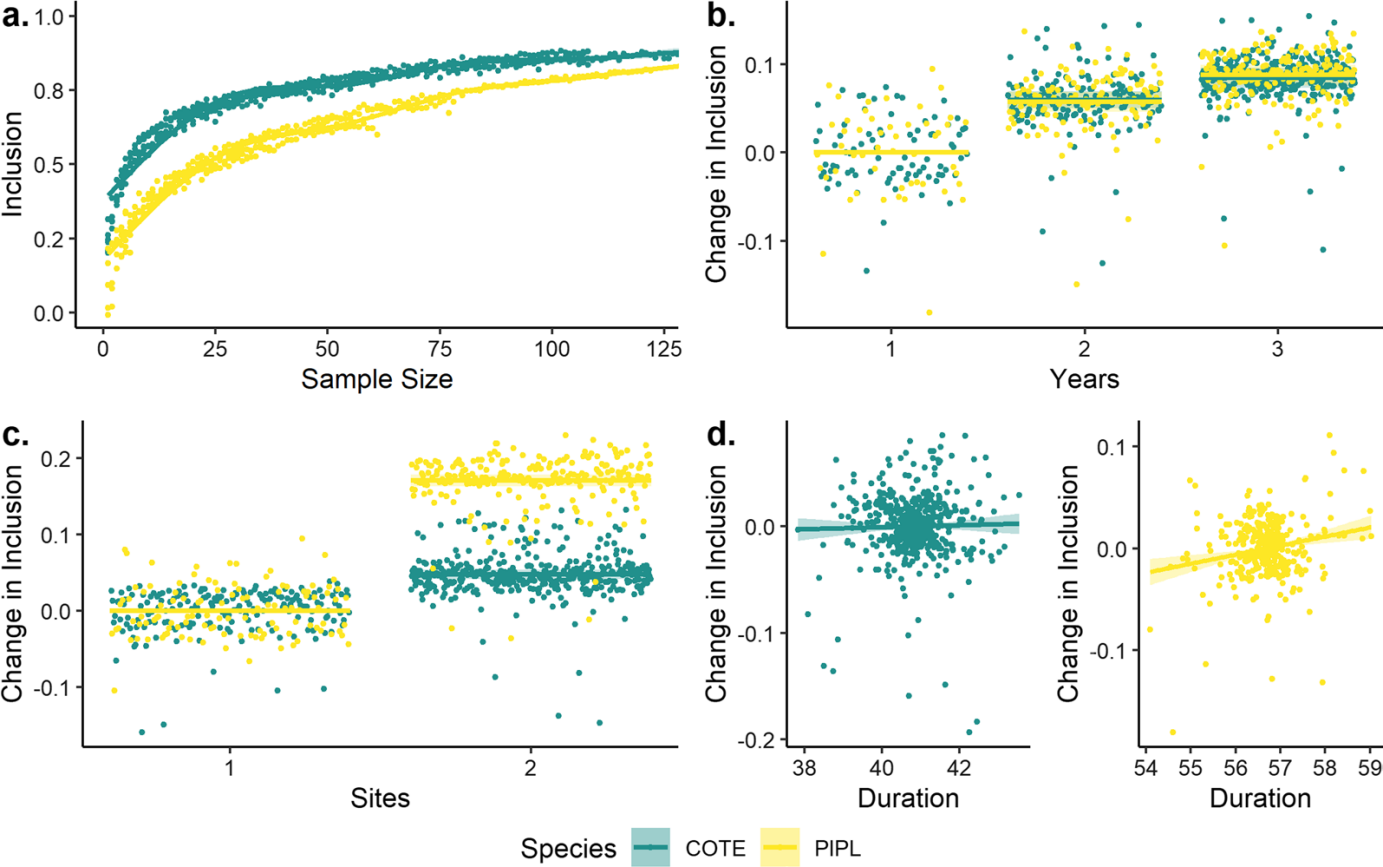
Partial effects plots for parameters included in generalized additive models of mean inclusion values for piping plovers (PIPL: 2015–2017, green) and common terns (COTE: 2014–2017, yellow): **a** sample size (conditional); **b** number of years (contrast to one year); **c** number of tagging sites (contrast to one year); and **d** mean duration of transmission (contrast). Inclusion values were significantly related (*p* < 0.001) to all parameters for both species except duration of transmission for common terns (*p* > 0.25)

**Fig. 4:**
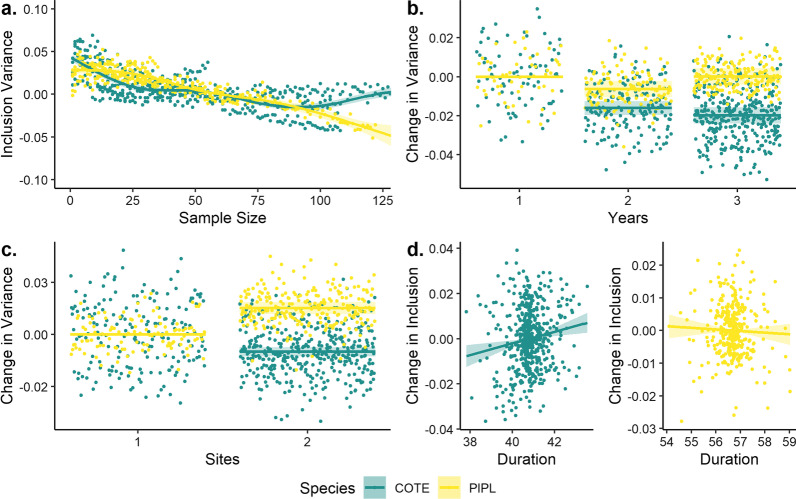
Partial effects plots for parameters included in generalized additive models of standard deviations of inclusion values for piping plovers (PIPL: 2015–2017, green) and common terns (COTE: 2014–2017, yellow): **a** sample size (conditional); **b** number of years (contrast); **c** number of sites (contrast); and **d** mean duration of transmission (contrast). Variance was significantly related (*p* < 0.001) to all parameters for both species except duration (*p* > 0.25, both species)

Both inclusion and variability were significantly related to sample size, year, and tagging site for both piping plovers and common terns, and of duration of transmission for piping plovers only (Figs. 3, 4). Baseline detection probabilities were greater for common terns than for piping plovers (Fig. 3a), while effects of additional sites and years were greater for piping plovers (Fig. 3b, c). Adding a second tagging site resulted in a 17% (CI = 16.3–17.8%) increase in inclusion compared to a single site for piping plovers, and a 5% (4.2–5.3%) increase for common terns (Fig. 3b). In comparison, adding a second year increased inclusion values less than adding a second site (+ 6% [4.7–6.8%] for both species) but more substantially than adding a third year (+ 3% [1.4–4.0%]) (Fig. 3c; Additional file 2: Fig. S1). Duration of transmission increased inclusion probability for piping plovers by 1% (0.4–1.4%) per day, but did not affect inclusion values for common terns (− 0.3 to 0.4%) (Fig. 3d). Number of detections per individual did not significantly affect inclusion values for either species (*p* > 0.25 for both).

In both species, sampling variance declined up to about 100 individuals (Fig. 4a). Variance among samples declined continuously in piping plovers but was stable or increasing above 100 individuals in common terns (Additional file 3: Fig. S2). Collecting a second and third year of data decreased variance by 0–0.5% in piping plovers and 1–2% in common terns relative to one year (Fig. 4b). Sampling two sites decreased variance by 0.8% (− 1.1 to − 0.6%) in common terns but increased variance by 1.3% (1.1–1.5%) in piping plovers relative to a single site (Fig. 4c). Duration of transmission increased variance for common terns (0.1–0.4%) but not piping plovers (− 0.2 to 0.1%; Fig. 4d), while number of detections per individual did not affect variance for either species.

**Fig. 5:**
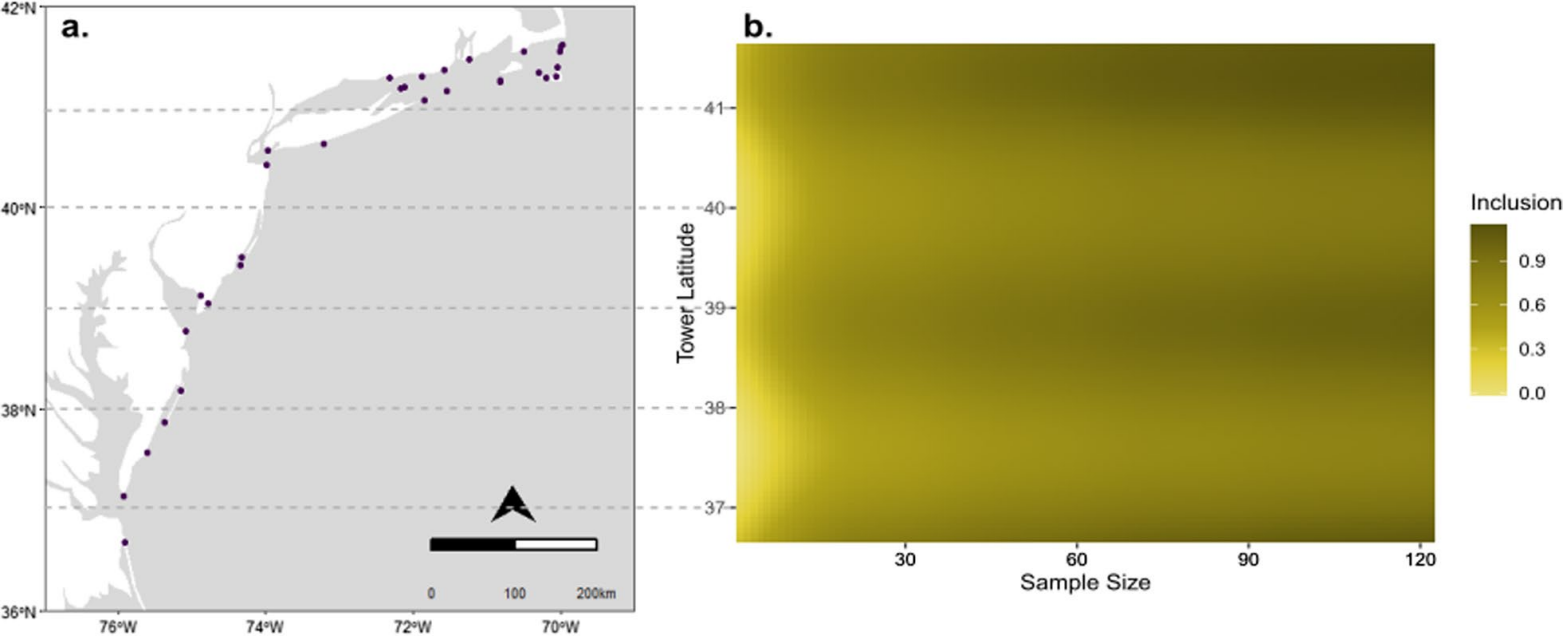
**a** Motus station tower locations (black dots), and **b** changes in tower-specific inclusion probabilities by latitude and sample size for piping plovers, 2015–2017. Darker colors represent higher probabilites of a tower being included in the sample

The probability of a station being included in a sample for piping plovers, given that tracked individuals were present at the station, was positively related to sample size, number of antennas, and the percent of the overall population using the station, and negatively related to the mean distance of the station from tagging locations (Additional file 4: Fig. S3). We found no relationship of station inclusion to the total number of detections at a station (i.e., length of stay of tracked birds in the vicinity of the station) or antenna altitude. Piping plovers were consistently detected at stations near tagging sites and at key stopover sites in Cape May, New Jersey and at the mouth of the Chesapeake Bay; inclusion probabilities decreased among these locations (Fig. 5).

## Discussion

Our bootstrapping analysis revealed an asymptotic relationship between sample size and inclusion probability for fixed receiving station sites, with the largest increase in representativeness of the unsampled population occurring over the first ~ 100 individuals. However, the magnitude and variability of this relationship depended on the species tracked, the distribution of transmitters among sites and years, the duration of transmission, and the migratory patterns of the target species, indicating that the target sample size and sampling strategy depend on the species and question of interest.

Across the two species included in our study, the representativeness of any given number of transmitters deployed was lower in piping plovers than in common terns within a single site or year, but higher across multiple sites and years. Piping plovers are relatively solitary, nesting diffusely along coastlines and exhibiting territorial behavior during non-breeding [10, 23], leading to potentially high variation among individuals in timing and direction of movement. In contrast, common terns are gregarious year-round, nesting in high densities at breeding colonies and staging and foraging in large flocks during non-breeding [11]. Although few studies directly compare sampling power between species with differing degrees of sociality, several anecdotal examples from marine megafauna suggest that relatively small samples may be sufficient to characterize behavior for species that migrate in groups [4]. In addition, any inter-individual variation in migratory routes of common terns, which migrate offshore, would have been less detectable in our land-based coastal station array than for near-shore-migrating piping plovers. However, the fact that common terns ultimately required a larger overall sample size to achieve equal levels of population-level representation in multi-site, multi-year samples suggests that, at the landscape scale, the patchier distribution of terns may ultimately require larger sample sizes to detect all groups. Finally, piping plovers had higher rates of non-detection than common terns (20% vs. 2%). While detections of common terns could be improved by placing receiving stations strategically near colonies and staging areas, the more diffuse distribution and higher individual variability in piping plover movements meant that the receiver network could not fully cover potential use areas. This suggests that a cushion for non-detection should be built into sample size considerations, particularly for species with sparse or unpredictable distributions or for which key habitat areas remain uncertain.

Effects of distributing transmitters among sites and years also varied by species. The increase in inclusion values from sampling multiple sites was substantially greater than from sampling multiple years in piping plovers. This suggests that the magnitude of spatial variation is greater than the magnitude of interannual variation in this population. In contrast, population-level variability in common tern movements was effectively represented by sampling a single breeding colony in the study region, with small improvements in representativeness and decreases in variability resulting from sampling across multiple sites or years. The relatively low interannual variability we observed in both species may be a result of high fidelity to breeding and wintering areas, which have been recorded for common terns [24, 25] and piping plovers [26, 27] in this region. Lower inter-site variability in common terns could result from overlap of individuals from different breeding areas at non-breeding sites, as is the case in this region [14, 25]. The non-breeding range of piping plovers is more diffuse [28–30], providing opportunities for segregation of individuals from different breeding sites during migration and wintering. Indeed, band resighting data suggest that piping plovers from nearby breeding areas may use different stopover sites and winter in different areas [8, 23]. Thus, our results indicate that species-specific variation in social behavior and migratory connectivity can impact not only required sample sizes, but also the optimal spatiotemporal distribution of capture locations to fully represent variation across the regional population.

We detected a positive effect of sampling duration on inclusion in piping plovers, but not common terns. In the context of this study, sampling duration represents the period during which tracked birds were detectable within our antenna array. Lack of detection may indicate transmitter loss or failure, bird movement outside the detection range of the station network, or mortality. Transmitter loss can be a considerable factor limiting data collection in studies using short term tag attachment methods aimed to minimize adverse effects, particularly when working with ESA-listed species. Only 47% (*n* = 70 of 150) of piping plovers tagged in southern New England were detected during migration. Field crews observed 25% (*n* = 37 of 150) of piping plovers with tag loss at breeding areas, suggesting that transmitter loss from our temporary attachment methods (i.e., glue to clipped feathers) was likely a leading cause of lost signals. Sampling duration of common terns is likely affected by tag loss and movement beyond detection range of land-based tracking stations. Common terns tagged using the glue and suture method have variable tag retention duration, ranging from < 1 month to 3 months [20].

Sampling duration of common terns and piping plovers is also limited by time spent within range of the station network and varied between the two species. Common terns use offshore migration routes [12, 13], which likely place them out of range of detection by land-based coastal antennas during migration. Correspondingly, detections of common tern generally ended at the conclusion of migratory staging, and space use across breeding and staging sites was relatively consistent throughout the detection period. However, piping plovers were detected further south along their fall migration routes, in keeping with their more nearshore migration strategies [7, 8]. Thus, increasing the length of the detection period also increased inclusion rates for more southerly receiving station sites used during migration in this species. The importance of transmission duration to detection of migratory stopover sites highlights both the need to design station arrays to capture seasonal movements of interest, as well as the importance of timing transmitter deployments to ensure that expected battery life and tag retention is sufficient to last through the intended period of data collection.

The probability of a used station being included in a sample varied along the migratory routes of piping plovers. Detection probabilities for stations used by at least 10% of tracked individuals were relatively consistent regardless of sample size, while less-used stations were frequently excluded, particularly at low sample sizes. Both frequency of use and inclusion probabilities for specific stations generally decreased with distance from the tagging site; however, the relationship was non-linear, and common staging areas were used and included more frequently than intermediate sites. This indicates that piping plovers may use direct routes over open ocean to travel between stopover areas rather than following the coastline throughout their migrations, which is consistent with patchy band resighting data for piping plovers during migration along the Atlantic coast [23] and modeled flight paths from detection data [8]. In addition, stations with 6 antennas were more likely to be included in the sample than those with 2–4 antennas regardless of sample size, station location, station altitude (although most stations were 10–12 m high, resulting in a relatively limited range of altitudes), or density of the target species. Our findings further suggest a negative relationship between the probability of detecting use of a specific site and the proportion of the population present at that site. Thus, sample size of tracked individuals required to assess fine-scale use of a given site (e.g., a wind energy lease area) may be higher for areas containing low densities of the target species, with rarer species requiring larger sample sizes to accurately detect and characterize movement. Our results emphasize the importance of designing receiving stations with sufficient numbers of antennas to detect species movements, as well as tailoring station networks to sample likely movement corridors.

Our bootstrapping analysis has several key limitations. Notably, population-level representation can only be evaluated in relation to the characteristics of the set of capture sites included. Thus, it is important to select tagging sites a priori to maximize representation across a relevant portion of the target species’ range. This may require foreknowledge of migratory routes and annual-cycle connectivity based on banding data and/or prior tracking. We were also able to compare effects of spatiotemporal sampling distribution over a relatively limited number of tagging sites (two) and years (three), which may or may not apply over larger numbers of sites or years. Since inclusion continued to increase across all sampling years, long-term studies are required to determine how many years of sampling are required to maximize inclusion values. Finally, our analysis is relatively simple in that we are only assessing baseline site occupancy. Evaluating more complex metrics (e.g., abundance, migratory routes, flight altitudes, habitat use), as well as associating changes in distribution with specific drivers, likely require longer durations and higher sample sizes than evaluating baseline occupancy patterns.

## Conclusions

Despite differences between species, our results highlight several consistencies in optimal study design. Both species required ~ 100–150 individuals to model 90% of used sites at the population level, with ~ 40–50 additional individuals needed to achieve 95% representativeness. The number of tracked individuals required to represent movement patterns at a single tagging site was similar regardless of location or species, as was the pattern of declining incremental improvement in representativeness for each additional sampling year. However, our results also highlight key differences between species, particularly the stronger benefits of distributing transmitters over multiple tagging sites in more solitary piping plovers compared to more gregarious common terns. We also found that effectiveness of automated radio telemetry to evaluate migration routes of piping plovers increased with sampling duration and was sensitive to receiving station design, placement, and intensity of use. Thus, while recommendations for sample sizes and temporal distribution of tag deployments may be similar across species and questions, geographic distribution of both tagging sites and VHF receiving stations is sensitive to differences among species and research questions.

While our study emphasizes the importance of carefully considering species-specific behavior and distribution to inform sample sizes and spatiotemporal transmitter distribution, baseline guidance for allocating transmitters could be implemented based on consistent patterns and then increased as needed to account for varying spatial concentrations, movement patterns, and questions of interest. Such baseline guidance could help to facilitate the rapid integration of individual movement into predicting and monitoring impacts of emerging habitat changes including offshore wind energy development. For example, our study suggests the information gained from any sample size increases when transmitters are divided among multiple sites and, to a slightly lesser extent, multiple years. A standard framework for gathering pre-construction data on presence and habitat use within a new offshore wind lease area might therefore specify a minimum sample size requirement (e.g., 100 transmitters) and require that the units be distributed among at least 2–3 different breeding sites, with at least two consecutive years of data per site. Such guidelines would help maximize the information value of each transmitter while providing robust, consistent sampling targets that are readily comparable among lease areas.

## Supplementary Information

**Additional file 1. Table S1: **Locations of receiving stations along the Atlantic coast that were active throughout the study period and detected tagged piping plovers (PIPL; 2015-2017) or common terns (COTE; 2014–2017).

**Additional file 2. Figure S1: **Change in inclusion mean with sample size conditional on a) number of years sampled, and b) number of sites sampled for piping plovers (2015–2017); and c) number of years sampled, and d) number of sites sampled for common terns (2014–2017).

**Additional file 3. Figure S2: **Change in standard deviation of inclusion mean with sample size conditional on a) number of years sampled and b) number of sites sampled for piping plovers (2015–2017); and c) number of years sampled, and d) number of sites sampled for common terns (2014–2017).

**Additional file 4. Figure S3: **Partial effects plots for parameters included in generalized additive models of tower-specific inclusion values for piping plovers, 2015–2017: a) mean distance to tagging sites (contrast); b) percent of population detected (contrast); c) number of detections per transmitter (contrast); and d) number of antennas per tower (contrast). Inclusion was significantly related (p < 0.001) to all parameters except detections per transmitter.

## Data Availability

The datasets supporting the conclusions of this article are available from the Motus website, (Project no.: 14, Project name: Loring, https://motus.org/data/downloads?projectID=14).

## References

[1] Hebblewhite M, Haydon DT (2010). Distinguishing technology from biology: a critical review of the use of GPS telemetry data in ecology. Philos Trans R Soc B.

[2] Rogers KB, White GC, Guy CS, Brown ML (2007). Analysis of movement and habitat use from telemetry data. Analysis and interpretation of freshwater fisheries data.

[3] Roberts A, Silverman E, Gifford S (2018). Sample size considerations for satellite telemetry and animal distributions. J Wildl Manage.

[4] Sequeira AM, Heupel MR, Lea MA, Eguíluz VM, Duarte CM, Meekan MG (2019). The importance of sample size in marine megafauna tagging studies. Ecol Appl.

[5] Girard I, Dussault C, Ouelette JP, Courtois R, Caron A (2006). Balancing number of locations with number of individuals in telemetry studies. J Wildl Manage.

[6] BOEM. 2022. Renewable energy GIS data [Internet]. Port Rowan, ON (Canada): Bureau of Ocean Energy Management; 2022. Available from: https://www.boem.gov/Renewable-Energy-GIS-Data/.

[7] Burger J, Gordon C, Lawrence J, Newman J, Forcey G, Vlietstra L (2011). Risk evaluation for federally listed (roseate tern, piping plover) or candidate (red knot) bird species in offshore waters: a first step for managing the potential impacts of wind facility development on the Atlantic Outer Continental Shelf. Renew Energy.

[8] Loring PH, McLaren JD, Goyert HF, Paton PWC (2020). Supportive wind conditions influence offshore movements of Atlantic Coast Piping Plovers during fall migration. Condor.

[9] Taylor PD, Crewe TL, Mackenzie SA, Lepage D, Aubry Y, Crysler Z (2017). The Motus wildlife tracking system: a collaborative research network to enhance the understanding of wildlife movement. Avian Conserv Ecol.

[10] Elliott-Smith E, Haig SM. Piping lover (*Charadrius melodus*), version 1.0. In: Billerman SM, editor. Birds of the world [Internet]. Ithaca: Cornell Lab of Ornithology; 2020. Available from: 10.2173/bow.pipplo.01.

[11] Arnold JM, Oswald SA, Nisbet ICT, Pyle P, Patten MA. Common Tern (*Sterna hirundo*), version 1.0. In: Billerman SM, editor. Birds of the world [Internet]. Ithaca: Cornell Lab of Ornithology; 2020. Available from: 10.2173/bow.comter.01.

[12] Goyert HF, Gardner B, Sollmann R, Veit RR, Gilbert AT, Connelly EE, Williams KA (2016). Predicting the offshore distribution and abundance of marine birds with a hierarchical community distance sampling model. Ecol Appl.

[13] Veit RR, White TP, Perkins SA, Curley S. Abundance and distribution of seabirds off Southeastern Massachusetts, 2011–2015. Sterling: U.S. Department of the Interior, Bureau of Ocean Energy Management; 2016. 82 p. Report No.: OCS Study BOEM 2016-067.

[14] Loring PH, Paton PWC, McLaren JD, Bai H, Janaswamy R, Goyert HF, Griffin CR, Sievert PR. Tracking offshore occurrence of common terns, endangered roseate terns, and threatened piping plovers with VHF arrays. Sterling, VA (USA): U.S. Department of the Interior, Bureau of Ocean Energy Management; 2019. 158 p. Report No.: OCS Study BOEM 2019-017.

[15] Birds Canada. motus: Fetch and use data from the Motus wildlife tracking system [Internet]. 2012. Available from: https://motusWTS.github.io/motus.

[16] Motus Receiver Locations [Internet]. Port Rowan, ON (Canada): Birds Canada; 2022 [updated 2022 May 31; cited 2022 May 31]. Available from: https://motus.org/data/receiversMap.

[17] Lascelles BG, Taylor PR, Miller MGR, Dias MP, Oppel S, Torres L (2016). Applying global criteria to tracking data to define important areas for marine conservation. Divers Distrib.

[18] Lascelles BG, Taylor PR, Miller MGR, Dias MP, Oppel S, Torres L, et al. Appendix 5: complete R Code from applying global criteria to tracking data to define important areas for marine conservation. 10.1111/ddi.12411.

[19] R Core Team. R: A Language and environment for statistical computing [Internet]. Vienna: R Foundation for Statistical Computing; 2016. Available from: https://www.R-project.org/.

[20] Loring PH. Evaluating digital VHF technology to monitor shorebird and seabird use of offshore wind energy areas in the Western North Atlantic [dissertation], Amherst: University of Massachusetts; 2016.

[21] Wood SN (2011). Fast stable restricted maximum likelihood and marginal likelihood estimation of semiparametric generalized linear models. J R Stat Soc.

[22] Wood S (2017). Generalized additive models: an introduction with R.

[23] Haig SM, Oring LW (1988). Mate, site, and territory fidelity in piping plovers. Auk.

[24] Egensteiner ED (1996). Site fidelity of common terns (*Sterna hirundo*) on Great Gull Island, New York [thesis].

[25] Bracey A, Lisovski S, Moore D, McKellar A, Craig E, Matteson S (2018). Migratory routes and wintering locations of declining inland North American common terns. Auk.

[26] Cohen JB, Fraser JD, Catlin DH (2006). Survival and site fidelity of piping plovers on Long Island, New York. J Field Ornithol.

[27] Noel BL, Chandler CR (2008). Spatial distribution and site fidelity of non-breeding piping plovers on the Georgia coast. Waterbirds.

[28] Nicholls JL, Baldassarre GA (1990). Winter distribution of piping plovers along the Atlantic and Gulf Coasts of the United States. Wilson Bull.

[29] Stucker JH, Cuthbert FJ, Winn B, Noel BL, Maddock SB, Leary PR (2010). Distribution of non-breeding Great Lakes piping plovers (*Charadrius melodus*) along Atlantic and Gulf of Mexico coastlines: ten years of band sightings. Waterbirds.

[30] Gratto-Trevor C, Haig SM, Miller MP, Mullins TD, Maddock S, Roche E, Moore P (2016). Breeding sites and winter site fidelity of piping plovers wintering in The Bahamas, a previously unknown major wintering area. J Field Ornithol.

