# Supplementary material for: Distributing transmitters to maximize population-level representativeness in automated radio telemetry studies of animal movement
Source: Mov Ecol. 2023 Jan 4;11:1. doi: 10.1186/s40462-022-00363-0 (PMC9814390; doi:10.1186/s40462-022-00363-0)
Supplement: Supplementary file 1 — Additional file 1. Table S1: Locations of receiving stations along the Atlantic coast that were active throughout the study period and detected tagged piping plovers (PIPL; 2015-2017) or common terns (COTE; 2014–2017). [file 40462_2022_363_MOESM1_ESM.docx]

Table S1. Locations of receiving stations along the Atlantic coast that were active throughout the study period and detected tagged piping plovers (PIPL; 2015-2017) or common terns (COTE; 2014-2017).

| Station | Lat | Long | # Antennas | COTE | PIPL |
| --- | --- | --- | --- | --- | --- |
| Parker River National Wildlife Refuge, MA | 42.780 | -70.808 | 3 | x |  |
| Race Point, Provincetown, MA | 42.066 | -70.244 | 6 | x |  |
| Monomoy Island (tern), MA | 41.620 | -69.985 | 6 |  | x |
| Monomoy Island (North), MA | 41.609 | -69.987 | 6 | x | x |
| Monomoy Island (South), MA | 41.553 | -70.010 | 6 | x | x |
| Waquoit Bay, Falmouth, MA | 41.552 | -70.507 | 6 | x | x |
| Sachuest Point, Newport, RI | 41.479 | -71.244 | 6 | x | x |
| Great Point, Nantucket, MA | 41.391 | -70.049 | 6 | x | x |
| Trustom Pond, RI | 41.373 | -71.576 | 6 | x | x |
| Muskeget Island, Nantucket, MA | 41.337 | -70.305 | 6 | x | x |
| Coatue Point, Nantucket, MA | 41.307 | -70.064 | 6 | x | x |
| Napatree Point, RI | 41.306 | -71.884 | 6 | x | x |
| Eel Point, Nantucket, MA | 41.293 | -70.197 | 6 | x | x |
| Great Island Natural Area, CT | 41.287 | -72.324 | 3 | x |  |
| Nomans Land (north), Martha's Vineyard, MA | 41.261 | -70.815 | 6 | x | x |
| Nomans Land (south), Martha's Vineyard, MA | 41.253 | -70.813 | 1 | x | x |
| Great Gull Island, NY | 41.202 | -72.119 | 4 | x | x |
| Plum Island, Buzzards Bay, MA | 41.189 | -72.163 | 6 | x |  |
| Block Island (southeast), RI | 41.153 | -71.553 | 6 | x | x |
| Montauk, NY | 41.072 | -71.856 | 6 | x | x |
| Fire Island, NY | 40.633 | -73.216 | 6 | x | x |
| Coney Island, NY | 40.574 | -73.977 | 4 | x |  |
| Sandy Hook, NJ | 40.430 | -73.985 | 6 | x | x |
| Rutgers Marine Field Station, Great Bay, NJ | 39.509 | -74.324 | 4 |  | x |
| North Brigantine Natural Area, NJ | 39.429 | -74.341 | 6 | x | x |
| Reeds Beach, Cape May, NJ | 39.127 | -74.891 | 3 | x |  |
| Wetlands Institute, Stone Harbor, NJ | 39.058 | -74.775 | 3 | x | x |
| Stone Harbor Point, NJ | 39.031 | -74.779 | 3 |  | x |
| Cape Henlopen State Park, DE | 38.770 | -75.085 | 6 |  | x |
| Assateague State Park, VA | 38.241 | -75.136 | 3 | x | x |
| Buntings, VA | 38.139 | -75.189 | 3 |  | x |
| Chincoteague National Wildlife Refuge, VA | 37.863 | -75.370 | 6 |  | x |
| Parramore Island Natural Area, VA | 37.574 | -75.617 | 4 |  | x |
| Skidmore Island, VA | 37.134 | -75.926 | 6 |  | x |
| Back Bay, VA | 36.672 | -75.916 | 6 | x | x |
