# Supplementary material for: Distributing transmitters to maximize population-level representativeness in automated radio telemetry studies of animal movement
Source: Mov Ecol. 2023 Jan 4;11:1. doi: 10.1186/s40462-022-00363-0 (PMC9814390; doi:10.1186/s40462-022-00363-0)
Supplement: Supplementary file 3 — Additional file 3. Figure S2: Change in standard deviation of inclusion mean with sample size conditional on a) number of years sampled and b) number of sites sampled for piping plovers (2015–2017); and c) number of years sampled, and d) number of sites sampled for common terns (2014–2017). [file 40462_2022_363_MOESM3_ESM.docx]

Figure S2. Change in standard deviation of inclusion mean with sample size conditional on a) number of years sampled and b) number of sites sampled for piping plovers (2015-2017); and c) number of years sampled, and d) number of sites sampled for common terns (2014-2017).


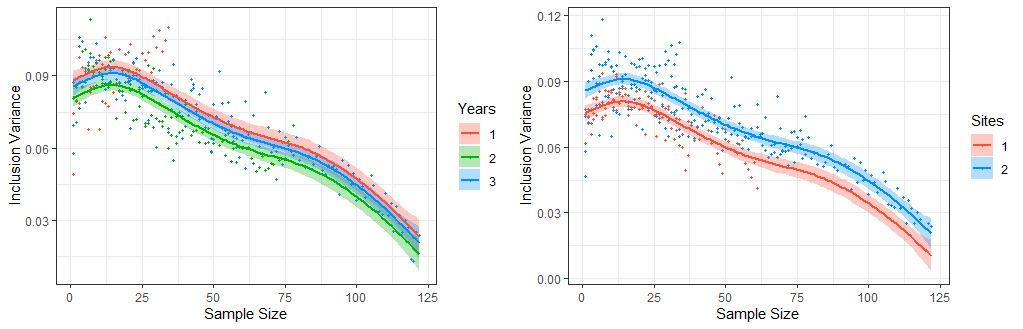


b.

a.


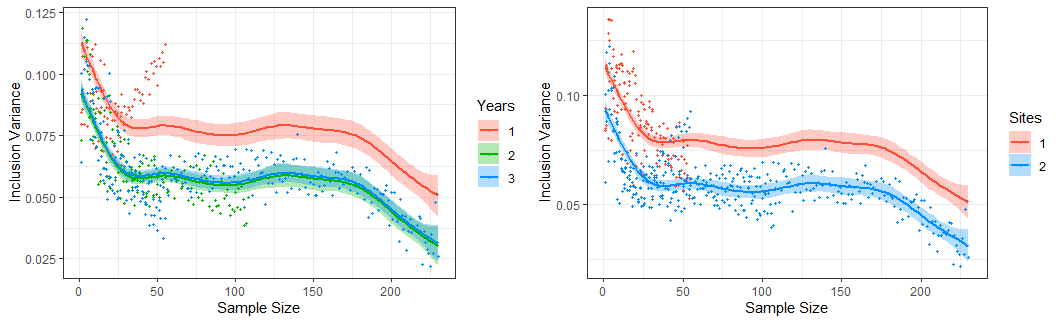


c.

d.
