# Supplementary material for: Distributing transmitters to maximize population-level representativeness in automated radio telemetry studies of animal movement
Source: Mov Ecol. 2023 Jan 4;11:1. doi: 10.1186/s40462-022-00363-0 (PMC9814390; doi:10.1186/s40462-022-00363-0)

Figure S3. Partial effects plots for parameters included in generalized additive models of tower-specific inclusion values for piping plovers, 2015-2017: a) mean distance to tagging sites (contrast); b) percent of population detected (contrast); c) number of detections per transmitter (contrast); and d) number of antennas per tower (contrast). Inclusion was significantly related (*p* < 0.001) to all parameters except detections per individual.


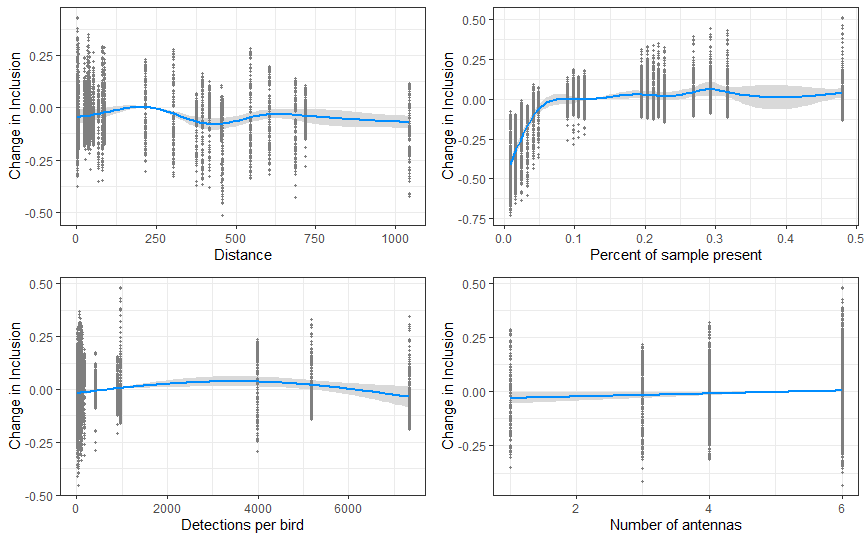

Supplement: Supplementary file 4 — Additional file 4. Figure S3: Partial effects plots for parameters included in generalized additive models of tower-specific inclusion values for piping plovers, 2015–2017: a) mean distance to tagging sites (contrast); b) percent of population detected (contrast); c) number of detections per transmitter (contrast); and d) number of antennas per tower (contrast). Inclusion was significantly related (p < 0.001) to all parameters except detections per transmitter. [file 40462_2022_363_MOESM4_ESM.docx]
